# “Location is surprisingly a lot more important than you think”: a critical thematic analysis of push and pull factor messaging used on Caribbean offshore medical school websites

**DOI:** 10.1186/s12909-017-0936-x

**Published:** 2017-06-02

**Authors:** Jeffrey Morgan, Valorie A. Crooks, Carla Jackie Sampson, Jeremy Snyder

**Affiliations:** 1 Department of Geography, Simon Fraser University, Burnaby, Canada; 2 Department of Public Affairs, University of Central Florida, Orlando, USA; 3 Faculty of Health Sciences, Simon Fraser University, Burnaby, Canada

**Keywords:** Medical school, Caribbean, Canada, Offshore medical school, Physician training, International medical education

## Abstract

**Background:**

Offshore medical schools are for-profit, private enterprises located in the Caribbean that provide undergraduate medical education to students who must leave the region for postgraduate training and also typically to practice. This growing industry attracts many medical students from the US and Canada who wish to return home to practice medicine. After graduation, international medical graduates can encounter challenges obtaining residency placements and can face other barriers related to practice.

**Methods:**

We conducted a qualitative thematic analysis to discern the dominant messages found on offshore medical school websites. Dominant messages included frequent references to push and pull factors intended to encourage potential applicants to consider attending an offshore medical school. We reviewed 38 English-language Caribbean offshore medical school websites in order to extract and record content pertaining to push and pull factors.

**Results:**

We found two push and four pull factors present across most offshore medical school websites. Push factors include the: shortages of physicians in the US and Canada that require new medical trainees; and low acceptance rates at medical schools in intended students’ home countries. Pull factors include the: financial benefits of attending an offshore medical school; geographic location and environment of training in the Caribbean; training quality and effectiveness; and the potential to practice medicine in one’s home country.

**Conclusions:**

This analysis contributes to our understanding of some of the factors behind students’ decisions to attend an offshore medical school. Importantly, push and pull factors do not address the barriers faced by offshore medical school graduates in finding postgraduate residency placements and ultimately practicing elsewhere. It is clear from push and pull factors that these medical schools heavily focus messaging and marketing towards students from the US and Canada, which raises questions about who benefits from this offshoring practice.

## Background

Students in the United States (US) and Canada have long travelled abroad for medical education. Although there are many reasons why these students choose to study abroad (e.g., international experience, familial ties), most do so because due to the competitiveness of domestic universities. For example, the Canadian Resident Matching Service (CaRMS) notes that some Canadians “opt to study medicine abroad because they have decided they would not be successful in Canada, or would rather not wait several years to be successful in their Canadian medical school application” ([1], p.6). Demand for undergraduate medical education has increased and there are considerably fewer training opportunities than there are strongly qualified applicants [2, 3]. As a result, determined students look abroad for the opportunity to study medicine. There are currently thousands of US and Canadian students are studying medicine abroad, many of whom are enrolled in what have come to be known as ‘offshore medical schools’ [1, 4, 5].

Offshore medical schools are for-profit, private educational enterprises located in the Caribbean region, purpose-built to provide undergraduate medical education to international students [6, 7]. We use the term Caribbean to signal the broader economic region, which includes countries in Central and South America that border the Caribbean Sea. Students typically spend 2 years studying basic sciences in the Caribbean, followed by 2 years studying clinical sciences in US hospitals. Though they accept students from around the world, most offshore medical schools heavily attempt to recruit US and Canadian students, both passively though their websites and actively through on-site information seminars [8, 9]. Graduates of these schools take qualifying exams for the country in which they wish to pursue postgraduate training and apply to residency positions as international medical graduates (IMGs). True to the ‘offshore’ business model, these medical schools operate outside of the training and licensing regulatory jurisdictions operating in the countries graduates wish to practice, such as the US or Canada, and can benefit from their close proximity to the US and Canada in terms of ease of travel and commonality of the English language [10, 11].

While the first offshore medical school opened in the 1970s, the industry has seen substantial growth in recent years, with dozens of offshore medical schools now operating across the Caribbean region and new schools opening each year [4]. This has created a highly competitive market. Despite dramatic growth in this offshore industry, little empirical research has been conducted about these medical schools [4]. Our current analysis draws on the content of offshore medical schools’ websites to provide insight into the discourses used to market these schools and attract future students. Having thoroughly reviewed the promotional content that populates the websites of Caribbean offshore medical schools, here we critically examine messaging on websites that both encourages prospective students to leave their home countries for medical education and argues as to why they should select a specific school in a particular country for their training, which we categorize as ‘push’ and ‘pull’ factors.

Literature examining the movement of people between countries for the purpose of delivering or receiving health care often uses a push-pull framework in order to identify and articulates the factors that inform decision-making and motivate some to ultimately go abroad [12]. We believe that a push–pull framework is similarly useful in understanding factors informing the decision-making of those considering moving between countries to attend medical school, which is why we employ it in the current study. Consistent with other research on migration and mobility, here push factors describe motivational desires or needs that are associated with the origin country, while pull factors are those which attract and are associated with the destination country [13]. Both push and pull factors typically operate simultaneously in informing a person’s decision to permanently or temporarily relocate, in addition to facilitating forces such as the absence of legal constraints [14]. As reported in this article, our thematic analysis is consistent with this, and reveals two push and four pull factors that are commonly included in the websites run by these institutions. The identification of these factors provides new insight into the entrepreneurial side of offshore medical schools, and specifically how they craft messages targeting potential applicants, aiding us in viewing these institutions as businesses that must market their services to compete for international customers.

There are numerous ways that prospective students can learn about offshore medical schools, such as through information sessions, word of mouth, and social media. We chose to focus on offshore medical school websites in this analysis because of the acknowledged and significant role that post-secondary school websites play in conveying admission and program information to potential students along with those already in attendance [15, 16]. As Saichaie ([17], pp.1–2) states:

Colleges and universities embrace the use of institutional websites due to their ability to rapidly communicate a significant amount of information to a potentially vast audience… In order to enhance identity and reputation, admissions and recruitment offices use language as a means to accomplish this end, with institutional websites serving as a primary outlet to represent these efforts.

Importantly, not only do these websites convey practical information but they can play a direct role in attracting prospective students [15]. As such, we view websites as a primary platform for offshore medical schools to communicate directly with, and compete for, prospective students. Thus, information and marketing on websites offer valuable new insight into our nascent understanding of this particular offshoring industry.

In the section that follows we provide details on the design of our analysis and our method for identifying websites for inclusion and gathering content. Following this we provide details on the push and pull factors that Caribbean offshore medical schools use to attract and compete for students identified in the thematic analysis, providing direct quotes throughout in order to enhance the reliability of what we report. In doing so we capture the true breadth of these factors, which include considerations as diverse as: tuition cost; physician demand in students’ home countries; education quality; and the promise of studying in a warm, tropical location. Finally, we move to critically reflect on the findings and consider what push and pull factors reveal about what is not being advertised, as well as suggestions for future research.

## Methods

In February and March, 2015 we compiled a comprehensive directory of English-language Caribbean offshore medical school websites, all of which are listed in Table 1. We took a triangulated approach to identifying offshore medical schools as we were aware that many existing listings were partial. We first identified a detailed listing of offshore medical schools on Wikipedia [18], which was compiled using multiple sources. We used this list as a starting point and contrasted it against an additional lists available on the student-run medical school information website StudentDoc [19] in order to locate schools not on the initial list. We next searched online for individual medical schools or directories for every Caribbean country, searching for information on each country separately, seeking to identify any offshore medical schools not captured by these two other lists. Following this, the third author (CS) then reviewed the list and contrasted it against one she had compiled separately over time through reviewing media and web sources to identify schools still not included, which resulted in the addition of one more school to the list. Our final step was to access the websites of every school on the master list we had gathered in order to remove any that were no longer in operation.

**Table 1:** Offshore medical school websites included in this analysis

Name of institution	Homepage ULR	Year founded	Country
All American Institute of Medical Sciences (AAIMS)	www.aaims.edu.jm	N/A	Jamaica
All Saints University School of Medicine - Dominica	www.allsaintsuniversity.org	2006	Dominica
All Saints University of Medicine - St. Vincent and the Grenadines	allsaintsu.org	N/A	St. Vincent and the Grenadines
American Global University School of Medicine	www.agusm.org	N/A	Belize
American International Medical University	www.aimu.us	N/A	St. Lucia
American International School of Medicine (AISM)	www.aism.edu	1999	Guyana
American University of Antigua (AUA) College of Medicine	www.auamed.org	2004	Antigua and Barbuda
American University of Barbados School of Medicine	www.aubmed.org	2012	Barbados
American University of Integrative Sciences - St. Maarten School of Medicine (AUIS)	www.auis.edu	1999	St. Maarten
American University of St. Vincent School of Medicine	www.ausmed.us	2012	St. Vincent and the Grenadines
American University of the Caribbean (AUC)	www.aucmed.edu	1978	St. Maarten
Atlantic University School of Medicine (AUSOM)	ausom.org [no longer operational]	N/A	St. Lucia
Aureus University School of Medicine	www.aureusuniversity.com	2004	Aruba
Avalon University School of Medicine (AUSOM)	www.avalonu.org	2003	Curaçao
Caribbean Medical University School of Medicine	www.cmumed.org	2007	Curaçao
Central America Health Sciences University	www.cahsu.edu	1996	Belize
College of Medicine and Health Sciences	www.comhssl.net	2001	St. Lucia
Georgetown American University (GUA)	www.gau.edu.gy	2013	Guyana
GreenHeart Medical University	www.greenheartmed.org	2004	Guyana
International American University College of Medicine (IAUCOM)	www.iau.edu.lc	2003	St. Lucia
International University of the Health Sciences (IUHS)	www.iuhs.edu	1997	St. Kitts and Nevis
International University School of Medicine (IUSOM)	www.internationaluniversity-schoolofmedicine.org	2005	Bonaire (additional campuses in Pakistan, Mexico, Colombia)
Medical University of the Americas	www.mua.edu	1998	St. Kitts and Nevis
Ross University School of Medicine	www.rossu.edu	1978	Dominica
Saba University School of Medicine	www.saba.edu	1986	Saba
Saint James School of Medicine Anguilla	www.sjsm.org	2001 (Bonaire) 2010 (Anguilla)	Anguilla
Saint James School of Medicine St. Vincent	www.sjsm.org	2014	St. Vincent and the Grenadines
Spartan Health Sciences University School of Medicine	www.spartanmed.org	1980	St. Lucia
St. George’s University School of Medicine	www.sgu.edu	1976	Grenada
St. Martinus University Faculty of Medicine	www.martinus.edu	2003	Curaçao
St. Matthew’s University School of Medicine	www.stmatthews.edu	1997	Cayman Islands
Texila American University (TAU)	www.tauedu.org	2010	Guyana
Trinity School of Medicine	www.trinityschoolofmedicine.org	2008	St. Vincent and the Grenadines
University of Health Sciences Antigua (UHSA)	www.uhsa.ag	1982	Antigua and Barbuda
University of Medicine and Health Sciences (UMHS)	www.umhs-sk.org	2007	St. Kitts and Nevis
University of Science, Arts and Technology - Montserrat	www.usat.edu	2003	Montserrat
Windsor University School of Medicine	www.windsor.edu	1998	St. Kitts and Nevis
Xavier University School of Medicine	www.xusom.com	2005	Aruba

Thirty-eight websites for unique English-language Caribbean offshore medical schools were identified and ultimately included in this analysis. An acknowledged limitation is that we have not included institutions that do not teach and advertise in English, though we are not sure if or how many such schools there are in operation. Despite our triangulated approach, another limitation is that the process we used to identify offshore medical schools may not have captured all those in operation. Finally, we did not attempt to verify whether the claims made on offshore medical school websites, particularly pertaining to students’ ability to practice in the US and Canada, were accurate. Table 1 contains a summary of the websites we identified, including name of institution, web address, location, and year founded. To the best of our knowledge, these 38 schools represent all English-language offshore medical schools in operation in the Caribbean at the time of the analysis; proposed or closed schools with websites were not included.

After an initial independent review of the 38 websites identified for this analysis and development of a summary of key information for each school (e.g., country of operation, admission requirements, number of graduates, etc.), JM and VAC met to identify analytic directions, at which point agreement was established regarding focusing on push and pull factors directed at intended students. Following this, JM visited every page of each website in order to extract explicit and implicit content pertaining to push and pull factors, using ‘sitemaps’ as a guide when available. After completing data extraction, the full spreadsheet was reviewed by JM and VAC in order to confirm the presence of discrete push and pull factor themes. Next, all authors independently reviewed the spreadsheet and, in light of the push and pull factor framework, came to consensus on six overarching themes that best summarize the factors articulated by these websites to compel students to consider training abroad (push) and why they should select a particular school (pull).

Upon identification of two push and four pull factors commonly articulated by Caribbean offshore medical schools when attempting to recruit potential students, our attention next turned to interpreting and understanding the meaning of these themes, as per the qualitative thematic analysis process [20]. To do this, our data extracts were coded by theme by JM, after which the coded extracts were independently reviewed by each team member. Together we discussed these extracts in order to come to consensus as to what each push or pull factor means, who these factors are intending to ‘speak’ to, relationships between these factors, and the full scope of each. We also considered the meaning of these factors in light of discussion in the scant existing literature about offshore medical schools, which is an important step in thematic analysis [20]. In the remainder of this paper we draw on these insights to investigate and critically discuss the scope and meaning of the push and pull factors identified in this analysis.

## Results

In reviewing the 38 offshore medical school websites (see Table 1), we identified two push and four pull factors that were commonly mentioned. We contend that the six push and pull factors examined here represent the foundational messages deployed by offshore medical schools to market themselves. In the remainder of this section we explore the scope and meaning of each of the push and pull factors identified in the thematic analysis, providing illustrative quotes to contextualize the findings. Although we present these factors separately below, we acknowledge that they are inherently related and mutually enforcing.

### Push factor: shortages of physicians in the US and Canada

Offshore medical school websites prominently feature information regarding physician shortages in the US and Canada. It is implied or explicitly stated that students interested in studying medicine should study in the Caribbean in order to become qualified to address physician shortages at home. For example, the website for Ross University School of Medicine in Dominica states: “*With the serious threat posed by a looming physician…shortage, Ross University’s mission of preparing highly trained doctors…has never been so critical…there will be a shortage of approximately 55,000 physicians in the US by 2020.*” In general, websites position offshore medical schools as a way to alleviate physician shortages. For example, the American University of Antigua dedicates a webpage to physician shortages in the US, suggesting that the offshore industry is a promising solution: *“Caribbean medical school graduates help fill the void of much needed primary care physicians… The realities of the physician shortage may seem bleak but by enrolling in a Caribbean medical school, you can be a part of the solution.*” Claims of physician shortage can appear multiple times on a given website, including in mission statements, frequently asked questions, or dedicated webpages. Discussion of physician shortage was limited to the US and Canada; there is no mention of physician shortages in any of the Caribbean countries where offshore schools are located, nor other source countries for international students (e.g., India, Pakistan, or Nigeria).

### Push factor: domestic schools are highly competitive

The competitive applicant pools for US and Canadian medical schools serve as a fundamental push factor behind the Caribbean’s offshore medical school industry. Statements positioning this competition as a rationale for seeking training abroad are common across the offshore medical school websites we reviewed. For example, the website for the Saint James School of Medicine, with campuses in St. Vincent and Anguilla, states:

*There has been no significant increase in the intake of U.S. medical schools in decades, while the number of qualified candidates increases every year. As a result, some students with the potential to become excellent physicians miss out on a medical education*.

Similarly, the The American University of Integrative Sciences St. Maarten School of Medicine states:

*Statistics show that…85 out of 100 Canadian Pre-Med students do not gain admission into medical school in Canada. Many students give up on their dream of becoming a physician. But you don’t have to! There is a completely reasonable and proven alternative to American and Canadian medical schools*.

These excerpts stress that offshore medical schools provide an opportunity for students who will otherwise “*miss out*” on medical education due to the competiveness of American and Canadian medical schools. Messages regarding competitive domestic medical school admissions are sometimes positioned alongside claims of physician shortages, suggesting an acknowledged relationship between these two push factors. For example, the website for the University of Medicine and Health Sciences in St. Kitts and Nevis states: “*Canada faces a growing shortage of physicians…[yet] more than 30,000 Canadian [medical school] applications are denied annually. The University of Medicine and Health Sciences offers an alternative path to becoming a MD in Canada.*”

### Pull factor: financial benefits

We found that offshore medical school websites typically make three common financial claims regarding the benefits of attending such schools: (1) competitive or low tuition rates; (2) the potential for access to government-backed student loans from students’ home countries; and (3) the possibility of private financing options. Reported tuition fees vary considerably, ranging from under US$50,000 to over US$250,000 for a four-year degree. These fees do not include textbooks, required supplies, or room and board in the Caribbean during classroom learning nor abroad during clinical rotations. For schools with low reported tuition rates, emphasizing cost-savings compared to medical school at home or other offshore medical schools in the region is a clear means to compete for prospective students. For example, the website for Avalon University School of Medicine located in Curaçao states that “*tuition fees are substantially lower than medical schools in Canada, [US] and other Caribbean medical schools”* while the International University of the Health Sciences in St. Kitts and Nevis suggests that its low tuition rates “*mean that you will not be burdened with large loans after graduation.*”

The second financial claim references the opportunity to secure federal or provincially-backed student loans, for US and Canadian students respectively. For example, the website for Ross University in Dominica states:

*The US Department of Education has certified [Ross University] as an eligible institution for Title IV Federal Direct Student Loans. [Ross University] students who qualify are eligible to receive US student loans in order to attend the university. [Ross University] is one of only four medical schools located in the Caribbean to earn this distinction.*

At the time the data were collected, students enrolled in one of only four offshore medical schools were eligible to receive US federal student loans. Perhaps unsurprisingly, these four schools also have some of the highest reported tuition fees. That said, there are reports that some schools encourage students to enroll in an online master’s program while concurrently enrolled in the MD program, in order to access student loans from their home countries [21, 22]. With regard to Canadian students, many of the websites reviewed include content aimed at Canadians who are eligible for provincially-backed student loans, claiming that these students are able to bring their loans abroad to their institutions. For example, the College of Medicine and Health Sciences in St. Lucia states: “*Canadian students who are interested in medical school may be able to receive Canadian Government financial support when attending the College of Medicine and Health Science, St. Lucia.”*

The third type of financial claim made on offshore medical school websites that relates to the overall pull factor of ‘financial benefits’ pertains to the availability of private financing options as a reason to attend specific offshore medical schools. Private financing includes the potential for scholarships or awards and assistance in securing private loans. For example, the All American Institute for Medical Sciences in Jamaica states:

*Students are eligible to apply for private educational loans from various private loan lenders. [Students] are eligible to apply on their own as long as they are credit worthy (no co-signer). It is highly recommended to apply with a co-signer. [The school’s] administration is committed to provide all the possible help from our end to facilitate the process for you*.

Other forms of assistance include payment schemes, which may make the process of financing a medical education more accessible. For example, the American International School of Medicine in Guyana outlines their payment plan on their website: “*Students can benefit from the AISM Payment Plan which requires an initial 100% payment on the first semester tuition which is paid one month prior to the commencement of the 1st semester.”*

### Pull Factor: geographic location and environment

Touristic discourse and images on offshore medical school websites are often deployed to highlight the beauty, if not even the exoticism, of the Caribbean region. Websites commonly use photo galleries with images of beaches and sunsets, alongside classrooms and laboratories, to convey a particular ‘Caribbean aesthetic.’ Accompanying text encouraged potential applicants to consider the promise of studying in a tropical setting. For example, the Caribbean Medical University School of Medicine, located in Curacao stated that its “*campus provides both a beautiful and comfortable environment for new experiences.”* Beyond characterizing the Caribbean as a beautiful place, some websites went as far as to suggest that the region is as a particularly attractive and pleasant environment in which to study medicine. As the American University of Antigua College of Medicine put it:

*Location is surprisingly a lot more important than you think. When you’re studying medicine in Connecticut, you’re probably not thinking about brutal winters or having to dig your car out of the snow. At a Caribbean medical school, you’ll be studying in a tropical paradise and that means no worries about crazy weather fluctuations. Seriously, winter is the worst*.

Websites are also quick to highlight close by tourist amenities and attractions. For example, the Saba University School of Medicine website states:

*Saba is a beautiful country—its nickname is the “Unspoiled Queen”— and is also extremely safe. Discriminating tourists have long sought out Saba for its diving, its restaurants, charming inns and stunning Caribbean vistas. Because Saba is small and off the well-worn tourist track, it lacks many of the distractions that can interfere with studying. Yet there is plenty to do, from hiking to deep sea diving.*

Interestingly, this excerpt paradoxically promotes the tourist activities available nearby, but uses the island’s lack of popularity among tourists as a point of competition, implying that students can be distracted at other offshore locations that have more prominent tourist sectors.

In addition to the potential of studying in the beauty of the Caribbean, the close geographic proximity of many offshore medical schools to the US and Canada is also an important aspect of the ‘geographic location and environment’ pull factor. For example, the website for St. Matthew’s University in Grand Cayman suggests potential flight plans:

*Grand Cayman is not only a beautiful location…it is also one of the safest… Grand Cayman is less than an hour’s flight from Miami, and also has direct flights from Atlanta, Chicago, Dallas, Charlotte, Houston, New York, Tampa, Toronto, and other international locations.*

These geographic pull factors present Caribbean offshore medical schools as a desirable and convenient location to study medicine, particular for US and Canadian students.

### Pull factor: training quality and effectiveness

We found that offshore medical school websites typically make three distinct claims regarding the quality and effectiveness of their medical training: (1) the rate of graduates passing medical licensing exams on their first attempt; (2) the presence of faculty trained in the US and Canada; and (3) the use of small class sizes. Offshore medical school websites make extensive reference to the United States Medical Licensing Examination (USMLE) which medical graduates – including those who trained internationally – are required to pass to practice in the US. In Canada, international medical graduates must pass the Medical Council of Canada Evaluating Examination (MCCEE), although reference to this exam appears much less frequently than to the USMLE. Offshore websites typically make reference to both graduates’ first time USMLE pass rate and curriculum that will prepare students to pass the exam. Some offshore medical school websites, like that of St. George’s University in Grenada, put pass rates in context:

*St. George’s 2012 performance on USMLE Step 1 was an improvement on the outstanding results from 2011, a year in which [the school’s] first-time test takers achieved a pass rate of 95% overall and 96% among those from the US and Canada. By contrast, the first-time taker pass rate for students at US and Canadian schools was 94% in 2011.*

Reported first time USMLE pass rates vary greatly across schools. For example, Spartan Health Sciences University of Medicine in St. Lucia reports a 70% USMLE Step 1 pass rate, while the International University of the Health Sciences reports “*Proven success: USMLE Step 1 pass rate of 87%.*”

Many offshore medical schools claim to have curriculum designed to prepare students for the USMLE exam. For example, the Avalon University School of Medicine in Curaçao states that the school’s “*program is designed for USMLE preparation and success. The curriculum is designed to incorporate USMLE style format questions in curriculum quizzes and exams.*” While websites make explicit claims regarding preparing students for passing the USMLE, some schools offer claims defending their commitment to quality by explaining that they do not simply train students for success on licensing exams. For example, the Xavier University School of Medicine in Aruba prominently states that its “*students typically score high on the USMLE, although we do not ‘teach for the test’.”*

Claims regarding the presence of faculty trained in the US and Canada are prominent on offshore medical school websites, and they intend to demonstrate the high quality of education. For example, the website for the University of Medicine and Health Sciences in St. Kitts and Nevis that its “*faculty is highly credentialed and recruited primarily from the United States. They love to teach and dedicate virtually 100% of their time to students.”* Although the presence of faculty members trained in Canada or the US seems to be an indication of quality, almost none of the sites distinguish between the diverse roles played by the faculty members listed on their websites (e.g., board members, teaching faculty, clinical faculty abroad, guest lecturers, visiting scholars, former instructors, laboratory supervisors, etc.), nor the start and end dates of their affiliations. This is significant because faculty members reported to have been trained in Canada or the US and listed on offshore medical school websites may in fact be those who oversee clinical placements abroad rather than those who actually teach on-site at the schools.

Small class sizes are frequently brought forward as a benefit to attending some offshore medical schools, and are framed as an incentive for seeking medical education at a small institution abroad. Many offshore schools that claim to have small class sizes make comparisons to other institutions with larger student bodies. For example, the website for the American University of St. Vincent School of Medicine states:

*With a small class size we are able to provide the much needed one on one attention to all the students. This makes for a more effective in-class, experience---you won’t find the anonymous, big lecture hall experience at [this school] the way you do at many schools.*

Similarly, the Aureus University School of Medicine in Aruba website suggests that small class sizes enable “*professors and staff…to provide you with a personalized educational experience as you work within your program.*” These quotes clearly frame small class size as an indication of the potential for high-quality and effective training at offshore medical schools.

### Pull factor: potential to practice in home country

As illustrated in the websites we reviewed, graduates’ abilities to return to Canada or the US to practice seems to be a central mandate of the offshore medical school industry. As such, claims regarding students’ abilities to return to their home countries for residency and to practice upon graduation are prolific and present across all offshore medical school websites. Schools generally reference students’ abilities to practice medicine in Canada or the US by making general statements about the experiences of former graduates. For example, the website for St. Matthew’s University in the Cayman Islands website states:

*Our graduates have earned residencies and/or permanent licensure in more than 40 states in the U.S., Canada, and numerous other countries. Our students achieve exceptional scholastic success, with U.S. licensing examination pass rates comparable to U.S. schools and well above the average of other non-U.S. schools.*

This quote is representative of the types of statements made across offshore medical school websites about the successes experienced by former graduates that are intended to attract future students. Such statements are noticeably absent from the sites of schools too young to have had a graduating class, in which case these schools focused on the potential for future graduates to ultimately practice abroad. In the US, several states, including California, Florida, and New York, have state-specific licensing requirements that do not include all offshore medical schools. As a result, the websites of offshore medical schools whose graduates cannot practice in all 50 states typically discussed practicing in the US in broad terms, while the few whose graduates are able to practice across the US made this explicitly clear on their websites. For example, the website for the American Global University School of Medicine in Belize, whose graduates are unable to practice in all 50 US states, asserts: “*In general all our graduates are eligible for licensure in the United States. Each State in the four territories that make up the United States have individual State licensing requirements.”* Meanwhile, the website for the American University of the Caribbean located in St. Maarten, whose graduates are approved to practice in all 50 states, proclaims: *“Some states, such as New York, California, and Florida, require approval for international medical schools. [This school] is proud to be approved in each of these states.”*

## Discussion

This analysis has brought forward and synthesized the dominant messages used by offshore medical schools in the Caribbean region on their websites to attract prospective students, and especially those from the US and Canada, which we classify as ‘push’ and ‘pull’ factors. While the two identified push factors were included on most of the websites we reviewed in this analysis, the four identified pull factors were more plentiful and were also more commonly raised. In the remainder of this section we examine themes that crosscut the push and pull factors identified and relate them to broader medical offshoring practices, identify critical contextual information that is missing from how these push and pull factors are discussed on offshore medical school websites, and propose important directions for future research on this topic.

### American and Canadian students are the clear focus of push and pull factors

It is clear from our findings that the push and pull factors presented on offshore medical school websites are oriented towards US and Canadian students. In fact, very few references were made to other origin countries from which students are known to leave to attend Caribbean offshore medical schools, such as India or Nigeria [23], on the websites we reviewed. Furthermore, despite the fact that these schools do attract small numbers of students from the Caribbean region [24], little effort was made to ‘speak to’ this local market on institutional websites. In other words, offshore medical school websites clearly identify Canadian and American students as those who are highly desirable and thus worthy of being marketed to via their websites. This was also reflected in the ubiquitous mentions of the USMLE across all websites, which is exclusively used in the US. Subtler references, such as featuring references to US and Canadian trained faculty, using the word ‘American’ or ‘America’ in the title (see Table 1), or comparing offshore medical school tuition fees to US tuition, are other ways in which offshore medical school websites signal to potential applicants that they are marketed towards students from these countries.

Because little research exists on offshore medical schools, it is difficult to say why offshore medical school advertisements are heavily directed at students from Canada and the US. Perhaps it is the fact that there is currently high demand for medical education among residents of these countries. Internationally-focused advertisement may also be a reflection of socioeconomic conditions in the Caribbean region, as high tuition fees and limited post-secondary opportunities can act as a barrier for local Caribbean students to attend medical school. It is also possible that the presence of students from the Global North is thought to lend credibility to these schools, as is the case with other globalizing phenomena such as in the practice of international physician voluntourism. International physician voluntourism often involves medical students from the Global North travelling to low-resource settings via ‘medical missions’ for clinical and research electives [25, 26]. As such, perhaps offshore medical schools similarly view students from these same Global North nations in high regard, and thus as a focus of online marketing. In other words, by marketing towards US and Canadian students, regardless of whether these students actually attend, offshore medical school websites signal a level of quality by association.

### Push and pull factors draw connections to health systems elsewhere

By heavily marketing themselves to potential US and Canadian students, positioning themselves as a solution to perceived physician shortages in these countries, and training students to do well on the USMLE, offshore medical school websites suggest an association with these countries’ health systems despite whether any formal relationship actually exists. In this way, offshore medical schools work to actively situate themselves as an extension of the US and Canadian medical training and healthcare systems, rather than those of Caribbean the nations in which they are located. We contend intentional marketing and positioning of offshore medical schools as extensions to the US and Canadian medical training and healthcare systems sets these offshore Caribbean medical schools apart from other international medical schools that US and Canadian students have a long history of attending, such as those in Ireland, Australia, and Eastern Europe [1, 27]. Further to this, medical schools conventionally exist to train physicians to serve the local population [28]. Some have characterized this as a social contract that medical schools enter with the public, exchanging dollars and subsidies to support biomedical and graduate research for benefits to public health [29, 30]. Meanwhile, it is clear from the marketing on offshore medical school websites that physicians are being trained to treat patients elsewhere. This sets offshore medical schools apart from other regional Caribbean medical schools, such as the University of the West Indies, that exist to train physicians to serve the needs of local populations [30]. In contrast, offshore medical schools exist to serve patient populations outside the Caribbean region, and our analysis shows that they are framing themselves as being a continuous part of a medical training and ultimately healthcare system in operation elsewhere.

Offshore medical schools share many similarities with other health-related offshoring practices or products operating in the Caribbean, such as medical tourism (i.e., the private movement of patients across international borders to purchase medical care). Specifically, both of these health care-related offshoring practices raise questions about if and how local populations actually benefit from the presence of these industries in the region [31]. With regard to the parallel offshoring sector of medical tourism, much research has suggested that this practice may encourage health workers in destination countries to cater to foreign patients instead of serving a local population, thereby exacerbating local health system inequities [32–34]. Similarly, while it is known that many Caribbean countries are facing physician shortages [35, 36], the push and pull factors we identified on offshore medical school websites do not reference that these schools are involved in addressing this local health human resources challenge by training physicians for local practice. Nor do they promote the potential for international graduates to aid in addressing this regional problem, while simultaneously encouraging US and Canadian students to train at these institutions to address physician shortages at home. Beyond not contributing to the supply of health workers in the Caribbean, these schools may actually exacerbate shortages when local physicians take up positions as lecturers at these schools or local students opt to enroll in order to ultimately establish a career in the US or elsewhere.

### Push and pull factors reveal competition between schools

Attempts to attract US and Canadian students via promotional websites, as well as intentionally positioning offshore medical schools as extensions to the US and Canadian healthcare systems, have produced an environment in which offshore medical schools seem to actively compete for students and tuition dollars, both against schools in the US and Canada and other offshore medical schools in the Caribbean. These competitive discourses, which as we discussed in the findings come through in these schools’ websites, convey a sense of the entrepreneurial nature of this offshoring industry and reveal that offshore medical schools are, in fact, businesses selling a product. This analysis shows that offshore medical schools, as illustrated through their websites, explicitly compete for students on a variety of points that are embedded in the push and pull factors they advertise. One clearly marketed point of competition is tuition fees, which are sometimes directly compared against competing institutions. For example, the Avalon University School of Medicine located in Curaçao states “*tuition fees are substantially lower than medical schools in Canada, [US] and other Caribbean medical schools.”* Tuition would certainly be of importance to prospective students, and research has established that such fees are critical to decision-making among university students [37–39], particularly in contexts in which offshore medical students are unable to secure government-backed student loans.

Discussions of first-time USMLE pass rates on offshore medical school websites reveal that this is another source of competition for students between schools, and especially those training students wanting to practice in the US. As we showed in the findings, many schools with strong pass rates promote this on their websites as a way to pull students to their campuses. However, what this rate does not show is that some Caribbean offshore medical schools reportedly allow only a subset of their students (i.e., high-achieving students) to write the USMLE Step 1 exam in order to maintain high first-time pass rates [21]. Such a practice demonstrates the importance of this comparative metric among offshore medical schools in attracting future students – a metric that has been criticized elsewhere for its inability to actually predict residents’ performance outside the exam [40, 41].

### What push and pull factors reveal about What’s not being advertised

We believe the push and pull factor lens we have used in the current analysis is useful for helping to understand offshore medical schools and the students who choose to attend them because it broadly encourages consideration of social, economic, and political conditions in both origin and destination countries that help us to understand why someone, including a student seeking medical training abroad, may choose to migrate [14, 42]. As Kline notes, the push–pull framework also maintains that, in addition to will, facilitating forces, such as the absence of legal constraints, must be in operation in order to enable movement [14]. Here lies a notable omission from offshore medical school websites: while no legal constraints impeded the migration of US and Canadian students to the Caribbean to study medicine, there are actually many de facto constraints that may impact their attempts to return home to practice. Unsurprisingly, mentions of these constraints are largely unaccounted for in the websites these schools use to market their training to international students and the push and pull factors we have identified here.

US and Canadian students who study abroad return as international medical graduates must compete for necessary residency placements alongside other international medical graduates [11, 43]. Currently, there are far fewer available postgraduate residency placements than there are applicants in both countries, especially in Canada [42]. For example, CaRMS reports that in 2013, only 499 international medical graduates matched to residency positions in Canada, a fraction of the 2962 international medical graduates that participated in the residency match [44]. Furthermore, 2013 was the first year “in CaRMS history in which more IMGs (2,962) participated in the match than [Canadian medical graduates] (2,837)” ([44], p.2). While it is unclear what proportion of these international medical graduates were Canadian or US citizens, it follows that as studying medicine abroad becomes increasingly popular the competition for these limited residency placements will also increase. As such, many offshore medical school graduates will be unmatched with residency placements and will not fulfill the promises laid out by these schools’ marketing messages, which suggest they can practice in their home countries and assist with lessening health worker shortages.

The possibility of American and Canadian students attending offshore medical school with the intent of practicing in one’s home country, and being told by these schools’ promotional websites that doing so is realistic, only to find out that residency placements are very limited in availability is particularly concerning given the high financial burden of attending an offshore medical school and reports of some offshore medical school graduates being left with unmanageable student debts [21]. For example, Canadians who studied medicine abroad had a median debt of CAD$160,000, compared to CAD$71,000 median debt held by graduates of domestic medical schools [45]. This represents a significant financial hardship without strong job prospects. As such, return migration after finishing medical school abroad, as suggested by pull factors advertised on Caribbean offshore medical school websites, may not be as unconstrained as it appears. Indeed, the journey to return home and practice after graduation is uncertain for many [46].

### Future research directions

This analysis brings forward new understanding into how offshore medical schools market themselves to prospective medical students using a ‘push’ and ‘pull’ framework commonly used in migration and mobility research [12]. We consider institutional websites to be a valuable source for understanding how offshore medical schools operate because post-secondary websites are known to greatly influence students’ choice of any particular institution [19], are a primary means to market to prospective students directly, and are readily accessible. There is ample opportunity for meaningful future research on this important topic, including using other sources of data. We outline some of these opportunities in this sub-section.

An important question for future research related to the current analysis is: what push and pull factors are brought forward by other marketing mediums, such as promotional posters or flyers, social media, and in-person information sessions? If meaningful differences are found in the push and pull factors used across different types of marketing materials, then these differences warrant examination and explanation. Drawing connections between the multiple marketing mediums used by offshore medical schools can assist in uncovering the complex networks at work to recruit potential students. Another available source of data about offshore medical schools are the readily accessible online blogs written by past, current, and future students. These blogs may reveal new insights into the push and pull factors identified here, including how offshore medical school students navigate the realities of these factors (e.g., studying in a tourist setting; coping with the practicalities of receiving student loans from home that can be used abroad; engaging in discussion with other students about the hopes and realities of being able to practice at home upon graduation). This analysis brings up wider, pressing research questions that relate to issues we have touched upon in the discussion. We believe that important questions include: what economic- and health-related impacts (both positive and negative) do offshore medical schools have on their Caribbean host countries; both from an ethical and practical perspective, should students’ home countries be playing any role in tracking students attending offshore medical schools, in providing them with financial support, and/or with reserving residency placements specifically for them; and what impacts do increasing numbers of offshore medical students actually have on the US and Canadian physician workforce, including addressing maldistribution.

## Conclusion

Offshore medical schools are private, for-profit enterprises located in the Caribbean region that provide undergraduate medical education primarily to international students, including many from the US and Canada. Often these students seek to return and practice in the US or Canada, or students from other countries use this training as a means to migrate to the US. This current thematic analysis reveals the dominant marketing messages presented on offshore medical school websites, which we characterize through an analytic lens of ‘push’ and ‘pull’ factors. We found two push and four pull factors were commonly mentioned across websites. Push factors include: the shortages of physicians in the US and Canada (targeted countries for applicants) that need to be addressed by training more medical professionals for practice in these countries; and the very low success rates of medical school applicants in Canada and the US that result in many with a desire to become a doctor unable to pursue training in these countries. Pull factors include the: financial benefits, such as competitive tuition rates, that can make some of these schools affordable to applicants; geographic location and environment of the Caribbean region, both of which can make studying abroad desirable; training quality, such as small class sizes that allow for personalized attention, and effectiveness, including track records of USMLE pass rates among graduates; and the potential for graduates to practice in their home countries after graduating from an offshore medical school. As these factors reveal, factors as diverse as one’s personal desire to be able to spend downtime on a tropical beach (as alluded to in the title of this article) to the collective moral responsibility to address physician shortages in one’s home country are being advertised to potential students as important reasons they should consider attending an offshore medical school in the Caribbean.

The push and pull factors identified in this analysis reveal three cross cutting themes that speak to how offshore medical schools portray themselves, and the students they market to, on their websites. First, it is clear that offshore medical school websites are heavily geared towards attracting applicants from the US and Canada. In fact, very few references are made to students from other regions attending these schools, let alone local students. Second, offshore medical school websites make a concerted effort to draw connections to health systems elsewhere, and actively work to situate themselves as an extension of the US and Canadian healthcare systems. Third, push-pull factors brought forward in this analysis reveal that offshore medical schools view themselves to be principally in competition with other offshore schools in the Caribbean region, as well as medical schools in the US and Canada. Each of these cross-cutting themes raise questions regarding who ultimately benefits from the offshoring practice of medical education, including the importation of students from the US and Canada, that has become a sizable industry in the Caribbean region. The push and pull factors identified in our analysis suggest that it is most likely not the citizens of these countries who are interested in pursuing medical education yet are almost completely invisible in the marketing messages used by these institutions nor the health systems of these same host nations or the wider Caribbean region, many of which are in significant need of trained health workers yet are not discussed as potential practice sites for graduates. These potential inequities warrant future research, as do most any issue relevant to offshore medical schools given how limited our state of knowledge is about these institutions.

## Abbreviations

CaRMS: Canadian Resident Matching Service
IMG: International Medical Graduate
MCCEE: Medical Council of Canada Evaluating Examination
MD: Doctor of Medicine
US: United States
USMLE: United States Medical Licensing Examination

## References

[1] Canadian Resident Matching Service (2010). Report on Canadians studying medicine abroad.

[2] Razack S, Hodges B, Steinert Y, Maguire M (2015). Seeking inclusion in an exclusive process: discourses of medical school student selection. Medical Educ.

[3] The Association of the Faculties of Medicine of Canada. Admission Requirements of Canadian Faculties of Medicine. 2015. https://afmc.ca/pdf/AdmissionRequirementsfor2015_en.pdf. Accessed 01 Feb 2017.

[4] Eckhert LN (2010). Private schools of the Caribbean: outsourcing medical education. Acad Med.

[5] Johnson K, Hagopian A, Veninga C, Hart LG (2006). The changing geography of Americans graduating from foreign medical schools. Acad Med.

[6] Ceaser M (2006). US exam boards urge scrutiny of offshore medical schools. Lancet.

[7] Shomaker S (2006). For-profit undergraduate medical education: back to the future?. Acad Med.

[8] American University of the Caribbean School of Medicine. Attend an AUC connect. 2016. http://www.aucmed.edu/admissions/get-started/open-house.aspx#Open%20House. Accessed 31 Aug 2016.

[9] St. George’s University. Information Sessions. 2016. http://www.sgu.edu/future-students/university-open-house.html. Accessed 31 Aug 2016.

[10] Ceaser M (2005). Offshore medical schools operate with minimal oversight. Chron High Educ.

[11] Babcock JM, Babcock BD, Schwartz MZ (2013). Maintaining a sufficient and quality physician workforce: the role of for-profit medical schools. Health Serv Insights.

[12] Walton-Roberts M (2014). International migration of health professionals and the marketization and privatization of health education in India: from push–pull to global political economy. Soc Sci Med.

[13] Prayag G, Ryan C (2011). The relationship between the 'push' and 'pull' factors of a tourist destination: the role of nationality – an analytical qualitative research approach. Curr Issues Tour.

[14] Kline DS (2003). Push and pull factors in international nurse migration. J Nurs Scholarsh.

[15] Adelman C (2006). How to design a web site that welcomes prospective applicants. Chron High Educ.

[16] Kittle B, Ciba D (2001). Using college web sites for student recruitment: a relationship marketing study. J Market High Educ.

[17] Saichaie K. Representation on college and university websites: an approach using critical discourse analysis. PhD Thesis. Univeristy of Iowa. 2011. http://ir.uiowa.edu/cgi/viewcontent.cgi?article=2456&context=etd. Accessed 31 Aug 2016.

[18] Wikipedia. List of Medical Schools in the Caribbean. 25 Jan 2017. https://en.wikipedia.org/wiki/List_of_medical_schools_in_the_Caribbean. Accessed 1 Feb 2017.

[19] StudentDoc. Caribbean Medical Schools. n.d. https://www.studentdoctor.net/2009/07/caribbean-medical-schools-a-good-option/. Accessed 1 Feb 2017.

[20] Vaismoradi M (2013). Content analysis and thematic analysis: implications for conducting a qualitative descriptive study. Nurse Health Sci.

[21] Halperin EC, Goldberg RB (2016). Offshore medical schools are buying clinical clerkships in U.S. hospitals: the problem and potential solutions. Acad Med.

[22] Lorin J. For-profit Caribbean medical schools use federal funds loophole. Bloomberg. Dec 2013. http://www.bloomberg.com/news/articles/2013-12-03/for-profit-caribbean-medical-schools-use-federal-funds-loophole. Accessed 31 Aug 2016.

[23] Deccan Chronicle. Caribbean islands lure Andhra Pradesh doctors away with good pay. 30 Jun 2014. http://www.deccanchronicle.com/140630/nation-current-affairs/article/caribbean-islands-lure-andhra-pradesh-doctors-good-pay. Accessed 31 Aug 2016.

[24] Barbados Nation News. American university wants more Barbadian students. 11 Nov 2015. http://www.nationnews.com/nationnews/news/74381/american-university-barbadian-students. Accessed 31 Aug 2016.

[25] Asgary R, Junck E (2013). New trends of short-term humanitarian medical volunteerism: professional and ethical considerations. J Med Ethics.

[26] Provenzano AM, Graber LK, Elansary M, Khoshnood K, Rastegar A, Barry M (2010). Short-term global health research projects by US medical students: ethical challenges for partnership. AmJTrop Med Hyg.

[27] Sullivan P (2000). Shut out at home, Canadians focking to Ireland's medical schools – and to an uncertain future. CMAJ.

[28] The Association of Faculties of Medicine of Canada. The Future of Medical Education in Canada. 2010. https://afmc.ca/pdf/fmec/FMEC-MD-2010.pdf. Accessed 31 Aug 2016.

[29] McCurdy L, Goode LD, Inui TS, Daughtery RM, Wilson DE, Wallace AG (1997). Fulfulling the social contract between medical schools and the public. Acad Med.

[30] The University of the West Indies. Statistical review academic year 2009/2010. 2009/2010. http://www.mona.uwi.edu/opair/statistics/2009-2010/UWI+Statistical+Review+2009-10.pdf. Accessed 31 Aug 2016.

[31] Adams K, Snyder J, Crooks VA, Hoffman L (2014). Medical tourism in the Caribbean: a call for cooperation. West Indian Med J.

[32] Johnston R, Crooks VA, Snyder J, Kingsbury P (2010). What is known about the effects of medical tourism in destination countries and departure countries?. A Scoping Review Int J Equity Health.

[33] Pocock NS, Phua KH (2011). Medical tourism and policy implications for health systems: a conceptual framework from a comparative study of Thailand. Singapore Malaysia Glob Health.

[34] Chen YY, Flood C (2013). Medical Tourism's impact on health care equity and access in low- and middle-income countries: making the case for regulation. J Law Med Ethics.

[35] Lofters AK (2012). The "brain drain" of health care workers: causes, solutions and the example of Jamaica. Can J Public Health.

[36] Pan American Health Organization (2008). Health systems profile Barbados.

[37] Himler MJ (1998). Post-secondary fees and the decision to attend a university or a community college. J Public Econ.

[38] Mazzarol T, Soutar GN (2002). "push-pull" factors influencing international student destination choice. IJEM.

[39] Wilkins S, Shams F, Huisman J (2013). The decision-making and changing behavioural dynamics of potential higher education students: the impacts of increasing tutition fees in England. Educ Stud.

[40] Bell JG, Kanellitsas I, Shaffer L (2002). Selection of obstetrics and gyencology residents on the basis of medical school performance. Am J Obstet Gynecol.

[41] Brothers TE, Wetherholt S (2007). Importace of the faculty interview during the resident application process. J Surg Educ.

[42] Arah OA, Ogbu UC, Okeke CE (2008). Too poor to leave, too rich to stay: developmental and global health coorelates of physician migration to the United States, Canada, Australia, and the United Kingdom. Am J Public Health.

[43] Curtis LJ, Dube U. Demographics, debt, and practice intentions of medical residents training in Canada. Can Public Policy. 2015:S138–49.

[44] Canadian Resident Matching Service (2013). 2013 R-1 main resident match report.

[45] Watts E, Davies JC, Metcalfe D (2011). The Canadian international medical graduate bottleneck: a new problem for doctors. Can Med Educ J.

[46] Barer ML, Evans RG, Hedden L (2014). Two wings and a prayer: should Canada make it easier for Canadian doctors trained abroad to enter practice here?. Healthc Policy.

